# Latent associations of low serum amylase with decreased plasma insulin levels and insulin resistance in asymptomatic middle-aged adults

**DOI:** 10.1186/1475-2840-11-80

**Published:** 2012-06-29

**Authors:** Toshitaka Muneyuki, Kei Nakajima, Atsushi Aoki, Masashi Yoshida, Hiroshi Fuchigami, Hiromi Munakata, San-e Ishikawa, Hitoshi Sugawara, Masanobu Kawakami, Shin-ichi Momomura, Masafumi Kakei

**Affiliations:** 1 First Department of Comprehensive Medicine, Saitama Medical Center, Jichi Medical University School of Medicine, 1-847 Amanuma, Omiya, Saitama, 330-8503, Japan; 2 Division of Clinical Nutrition, Department of Medical Dietetics, Faculty of Pharmaceutical Sciences, Josai University, 1-1 Keyakidai, Sakado, Saitama, 350-0295, Japan; 3 Department of Internal Medicine, Social Insurance Omiya General Hospital, 453, Bonsai, Kit, Japan; 4 Department of Health Care Center, Social Insurance Omiya General Hospital, 453 Bonsai, Kita, Saitama, 331-0805, Japan

**Keywords:** Serum amylase, Obesity, Body mass index, Insulin resistance, Leptin, 75-g oral glucose tolerance test, HOMA-R, HOMA-β, QUICKI

## Abstract

**Background:**

Low serum amylase is likely to be associated with obesity and metabolic abnormalities, which are often accompanied by impaired insulin action. However, it is unclear whether low serum amylase is associated with impaired insulin action in clinical settings. Therefore, we investigated the associations of low serum amylase with plasma insulin levels, and obesity-related parameters, including leptin.

**Research design and methods:**

We measured serum amylase, plasma insulin, obesity-related parameters such as leptin, cardiometabolic risk factors, and anthropometric parameters in a cross-sectional study of 54 asymptomatic subjects (mean age 48.6 ± 7.6 years) who were not being treated for diabetes.

**Results:**

Body mass index (BMI) and plasma glucose at 120 min after a 75-g oral glucose tolerance test (OGTT) were significantly higher in subjects with low serum amylase (< 60 IU/l, n = 21) than in those with normal-to-high serum amylase (n = 33) (*P* = 0.04 and *P* = 0.004, respectively). In univariate correlation analysis, serum amylase was significantly correlated with BMI alone (*r* = –0.39, *P* = 0.004). By contrast, multivariate logistic analysis showed that each 1-SD increase in quantitative insulin sensitivity check index, and each 1-SD decrease in plasma insulin OGTT at 0 and 60 min, homeostasis model assessment of insulin resistance (HOMA)-R, and HOMA-β were significantly associated with low serum amylase, particularly after adjusting for BMI. When subjects were divided into three groups according to HOMA-R, serum amylase levels were significantly lower in subjects with HOMA-R > 2.5 (n = 23) compared with subjects with HOMA-R 1.6–2.5 (n = 10) (61.1 ± 13.6 U/ml versus 76.9 ± 20.5 U/ml, Bonferroni test, *P* = 0.02), but not compared with subjects with HOMA-R<1.6 (n = 21; 62.7 ± 17.6 U/ml). Similar trends were observed when subjects were divided according to plasma leptin and fasting plasma insulin levels.

**Conclusions:**

These results suggest that after adjusting for BMI, low serum amylase is associated with decreased basal insulin levels and insulin secretion, as well as high insulin resistance. The nature of these associations remains to be elucidated in further studies.

## Introduction

For many years, low serum amylase was thought to reflect diffuse pancreatic destruction secondary to advanced pancreatic diseases, such as chronic pancreatitis [1,2]. Recently, several large clinical studies have shown that low serum amylase is also associated with metabolic syndrome and diabetes [3,4]. However, the mechanisms underlying these associations remain unclear. In previous epidemiological studies [3,5], we observed a marked negative association between serum amylase and body mass index (BMI). Consequently, we hypothesized that high BMI (i.e., obesity) is the most critical factor that is inversely correlated with serum amylase, and that insulin inactivity is a putative secondary factor that regulates the observed association. In our previous sub-analysis [5], we found that the relationship between serum amylase levels and glycated hemoglobin (HbA1c) was rather complicated (not a simple linear relationship), particularly in individuals with normal or mildly impaired glucose metabolism. Unfortunately, plasma insulin and obesity-related parameters, such as leptin and tumor necrosis factor (TNF) α, were not measured in the earlier studies. Therefore, we were unable to determine whether impaired insulin action, particularly insulin resistance, could mediate the associations between low serum amylase and diabetes and metabolic syndrome.

In this cross-sectional study of asymptomatic middle-aged subjects without advanced diabetes, plasma insulin and obesity-related parameters were measured along with other cardiometabolic risk factors. Here, we report latent associations between low serum amylase and plasma insulin, insulin resistance, and obesity-related parameters. Leptin, an adipokine that regulates energy balance through a wide range of systemic functions, is associated with obesity and insulin resistance [6,7]. Additionally, TNFα, a proinflammatory adipokine, was proposed as a link between obesity and insulin resistance [8,9]. Therefore, we also examined the associations between low serum amylase and these adipokines in this study.

## Methods

### Subjects

This study was based on a composite research program that was conducted in collaboration with Saitama Medical Center, Jichi Medical University School of Medicine, Saitama, Japan, and the Social Insurance Omiya General Hospital, Saitama, Japan. As in the previous study by [Aoki et al. 10], we recruited subjects who visited Social Insurance General Omiya Hospital between October 2008 and December 2009, for annual metabolic health checkups. Exclusion criteria were as follows: taking any antihypertensive or hypoglycemic agent, diuretic, lipid-lowering agent or insulin; and history of malignancy, liver disease or kidney disease. Subjects lacking data for serum amylase and responses to the lifestyle questionnaire were also excluded. Consequently, 44 men and 10 women were included in the current analysis. The study was approved by the Ethics Committee at Jichi Medical University, Tochigi, Japan. All subjects were informed about the study and provided written consent. Anthropometric measurements and laboratory tests were conducted using standard methods as previously described [10]. Blood samples were obtained in the early morning after an overnight fast. All of the subjects then underwent a 75-g oral glucose tolerance test (OGTT), and blood samples were obtained at 60 and 120 min to measure plasma glucose (PG) and plasma insulin (PI) concentrations. Insulin, leptin, and TNFα were measured using commercially available enzyme-linked immunosorbent assays (insulin: Yanaihara Institute Inc., Shizuoka, Japan; leptin: R&D Systems, Minneapolis, MN, USA; TNFα: Invitrogen Corp, Carlsbad, CA, USA).

Homeostasis model assessment of insulin resistant (HOMA-R), an index of insulin resistance, quantitative insulin sensitivity check index (QUICKI), and HOMA-β, an index of the insulin secretion capacity in the basal state, were calculated using the following equations:

(1) $$\text{HOMA}-\text{R}=\left(\text{FPG}\times \text{FPI}\right)/405$$

(2) $$\text{QUICKI =}\;1/\left[\text{log}\left(\text{FPI}\right)\;\text{+ log}\;\left(\text{FPG}\right)\right]$$

(3) $$\text{HOMA}-\beta =(360\times \text{FPI})/\left(\text{FPG}-63\right)$$

where FPG = fasting plasma glucose and FPI = fasting plasma insulin.

Serum amylase levels were measured using an enzymatic method (L-type Amylase assay; Wako, Tokyo, Japan) with a normal range of 41–112 IU/l, a detection limit of 1.7 IU/l, and a run-to-run coefficient of variation < 5.0%. PG was measured by the glucose oxidase method. HbA1c was measured in Japan Diabetes Society (JDS)-HbA1c units by high-performance liquid chromatography. HbA1c was converted to National Glycohemoglobin Standardization Program (NGSP) levels by the formula HbA1c (%) (NGSP) = HbA1c (JDS) (%) + 0.4%, considering the relational expression of HbA1c (JDS) (%) measured by the previous Japanese standard substance and measurement methods [11]. Since serum amylase levels can be affected by kidney function because of its excretion from the kidney [12,13], the estimated glomerular filtration rate (eGFR) was included as a confounding factor in multivariate logistic analysis. eGFR was calculated using the Modification Diet in Renal Disease study equation for Japanese subjects [14], as follows:

(4) $$\text{eGFR}\left(\text{ml}/\text{min}/1.73\;{\text{m}}^{2}\right)=194\times {\text{serum Cr}}^{-1.094}\times {\text{age}}^{-0.287}\left(\text{if female}\right)\times 0.739$$

where Cr = serum creatinine concentration (mg/dl).

### Statistical analysis

All data are expressed as means ± SD or median (interquartile range). Subjects were dichotomized according to the serum amylase level as either low (< 60 IU/l, n = 21) or high (≥ 60 IU/l, n = 33), because the 25^th^ percentile of serum amylase level was approximately 60.0 IU/l in our large epidemiological study [3], in which serum amylase concentration was measured using the same methods at our hospital. Since the present study involved a small number of subjects, we used the earlier data to estimate the threshold for low serum amylase. *P*-values for continuous variables and categorical variables were determined using the Mann–Whitney test and the *χ*^2^-test with Yates’s correction, respectively. Because the distribution of triglyceride, PI at 0, 60, and 120 min, HOMA-R, HOMA-β, leptin, and TNF were highly skewed, these values were log-transformed before analysis. To examine the univariate linear correlations among serum amylase and variables associated with insulin resistance and glucose metabolism, Pearson’s correlation coefficients were determined. Multiple stepwise regression analysis was conducted to identify independent parameters that significantly explained serum amylase levels. Categorical dichotomous parameters were labeled as 0 or 1. In multivariate logistic regression models, the odds ratios for low serum amylase were calculated for each 1-SD increase/decrease in the clinical variables. To examine the effects of insulin resistance, subjects were divided into three groups according to HOMA-R, leptin levels, and PI during OGTTs. Normal, moderate and high HOMA-R were defined as < 1.6, 1.6–2.5, and > 2.5, respectively, according to studies by [Taniguchi et al. 15] and the criteria proposed by the Japan Diabetes Society 2010. The numbers of subjects in the three groups (normal, moderate, high) of leptin levels and PI during OGTTs were set to be similar to those in the normal, moderate, and high HOMA-R groups, because the thresholds for these values have not been established. Serum amylase was examined by two-way analysis of variance (ANOVA) with HOMA-R, leptin, and PI during OGTTs as one factor, and obesity (i.e., BMI) as the second factor. Significant differences in serum amylase between groups were determined using Bonferroni’s *post hoc* test and *P*<0.017 was considered statistically significant. Statistical analysis was performed using SPSS software version 18.0 (SPSS-IBM Chicago, IL, USA). Values of *P*<0.05 were considered statistically significant, except in the *post hoc* tests following ANOVA.

## Results

The mean and median values of most clinical parameters were within the normal ranges (Table 1). Five subjects were suspected of having type 2 diabetes because their PG at 120 min was ≥ 200 mg/dl, even though FPG was<126 mg/dl. BMI and PG at 120 min were significantly higher in subjects with low serum amylase than in those with normal to high serum amylase. Table 2 shows the univariate linear correlation coefficients between serum amylase and variables associated with insulin resistance and glucose metabolism. Serum amylase was significantly correlated with BMI, but no other variable. HOMA-R was very closely correlated with FPI (*r* = 0.997, *P*<0.001), but showed weaker correlation with FPG (*r* = 0.53, *P*<0.001, data not shown). Stepwise regression analysis revealed that of the independent clinical variables listed in Table 1, BMI and PI at 60 min (β coefficient = –0.56 and 0.38, respectively, adjusted R^2^ = 0.26, data not shown) were significantly associated with serum amylase. In multivariate logistic analysis, each 1-SD increase in BMI was significantly associated with low serum amylase, even after controlling for confounding factors (Table 3). By contrast, each 1-SD increase in QUICKI, and 1-SD decreases in PI at 0 and 60 min, HOMA-R, and HOMA-β was significantly associated with low serum amylase, particularly after adjusting for BMI (** *Model 3* **). The significant association between low serum amylase and PG at 120 min disappeared after adjusting for BMI. In all analyses adjusted for BMI (** *Model 3* **), each 1-SD increase in BMI was significantly associated with low serum amylase (data not shown).

**Table 1:** Clinical characteristics of subjects divided by serum amylase levels

	**Low serum amylase (< 60 IU/ml)**	**Normal to high serum amylase (≥ 60 IU/ml)**	***P* values**
n	21	33	–
Age	47.1 ± 7.3	49.6 ± 7.8	0.22
Men, n (%)	17 (81.0)	27 (81.8)	0.93
Body mass index (kg/m^2^)	25.5 ± 3.9	23.4 ± 2.6	0.04
Systolic blood pressure (mmHg)	119 ± 18.5	118 ± 15.0	0.88
Diastolic blood pressure (mmHg)	71.9 ± 11.6	73.8 ± 11.5	0.54
Triglyceride (mg/dl)	117 (88-184)	92 (76-125)	0.17
HDL-cholesterol (mg/dl)	55.9 ± 16.1	54.8 ± 10.7	0.96
Creatinine (mg/dl)	0.8 ± 0.2	0.8 ± 0.1	0.93
eGFR (ml/min/1.73 m^2^)	84.8 ± 16.9	80.5 ± 11.1	0.56
Amylase (IU/l)	49.4 ± 7.6	74.6 ± 14.1	—
(range)	(32–59)	(60–121)	
HbA1c (%) (NGSP)	5.7 ± 0.3	5.6 ± 0.3	0.47
FPG (mg/dl)	102 ± 11	98 ± 7.6	0.32
PG at 60 min (mg/dl)*	144 ± 40	138 ± 33	0.56
PG at 120 min (mg/dl)*	151 ± 44	119 ± 35	0.004
FPI (μU/ml)	6.0 (3.6–14.9)	10.3 (5.1–13.1)	0.30
PI at 60 min (μU/ml)*	32.2 (18.7–62.1)	55.3 (41.7–86.3)	0.06
PI at 120 min (μU/ml)*	47.3 (31.6–102)	45.4 (29.0–65.6)	0.67
HOMA-R	1.5 (0.9–3.9)	2.3 (1.2–3.4)	0.45
QUICKI	0.38 ± 0.10	0.34 ± 0.04	0.45
HOMA-β	57.6 (34–125)	104 (54-138)	0.10
Leptin (pg/ml)	37.3 (18–55)	27.9 (18–43)	0.40
TNFα (pg/ml)	0.69 (0.5–1.1)	0.56 (0.5–0.7)	0.08
Type 2 diabetes^†^	4	1	0.13
Current smokers, n (%)	6 (28.6)	11 (33.3)	0.95
Everyday alcohol drinkers, n (%)	10 (47.6)	9 (27.3)	0.19
Regular exerciser, n (%)	3 (14.3)	7 (21.2)	0.78

Data are means ± SD or medians (interquartile range) for highly skewed variables.

Continuous variables and categorical valuables were compared using the Mann–Whitney test and *χ*^2^-test, respectively.

*Measured during a 75-g OGTT.

^†^Five subjects were suspected of having type 2 diabetes because PG at 120 min was ≥ 200 mg/dl.

*eGFR*: estimated glomerular filtration rate; *FPG*: fasting plasma glucose; *FPI*: fasting plasma insulin; *HDL-C*: high-density lipoprotein; *HOMA*: homeostasis model assessment; *OGTT*: oral glucose tolerance test; *NGSP*; National Glycohemoglobin Standardization Program; *PG*: plasma glucose; *PI*: plasma insulin; *QUICKI*: quantitative insulin sensitivity check index; regular exerciser: subjects who exercise over 30 minutes at least twice per week; *TNF α*: tumor necrosis factor α.

**Table 2:** Correlations between serum amylase and BMI with variables associated with insulin resistance and glucose metabolism

	**Amylase**	**BMI**
HOMA-R	–0.006	0.47***
QUICKI	–0.08	–0.33*
HOMA-β	0.08	0.44***
Leptin	–0.11	0.50***
TNFα	–0.18	0.24
FPI	0.01	0.47***
PI at 60 min^†^	0.13	0.45***
PI at 120 min^†^	–0.09	0.50***
FPG	–0.23	0.28
PG at 60 min^†^	–0.16	0.43**
PG at 120 min^†^	–0.27	0.34*
HbA1c	–0.13	0.43**
BMI	–0.39**	

Pearson’s correlation coefficients are shown.

HOMA-R, HOMA-β, leptin, TNFα, FPI, PI at 60 min, and PI at 120 min were log-transformed before analysis.

*P<0.05, **P<0.01, ***P<0.001.

^†^Measured during a 75-g OGTT.

*BMI*: body mass index; *FPG*: fasting plasma glucose; *FPI*: fasting plasma insulin; *HOMA*: homeostasis model assessment; *OGTT*: oral glucose tolerance test; *PG*: plasma glucose; *PI*: plasma insulin; *QUICKI*: quantitative insulin sensitivity check index; *TNFα*: tumor necrosis factor α.

**Table 3:** Associations between clinical variables and low serum amylase

	1-SD*		**Odds ratio (95% CI)**	***P* value**
BMI	Inc	Model 1	2.22 (1.06–4.67)	0.03
		Model 2	4.41 (1.36–14.3)	0.01
HbA1c	Inc	Model 1	1.30 (0.74–2.29)	0.35
		Model 2	1.39 (0.75–2.59)	0.29
		Model 3	0.80 (0.37–1.75)	0.57
FPG	Inc	Model 1	1.55 (0.87–2.75)	0.13
		Model 2	1.46 (0.72–2.94)	0.29
		Model 3	1.12 (0.51–2.49)	0.77
PG at 60 min^†^	Inc	Model 1	1.18 (0.68–2.06)	0.55
		Model 2	1.10 (0.56–2.18)	0.78
		Model 3	0.58 (0.23–1.43)	0.77
PG at 120 min^†^	Inc	Model 1	2.45 (1.20–4.99)	0.01
		Model 2	2.55 (1.12–5.80)	0.03
		Model 3	1.78 (0.74–4.27)	0.20
FPI	Dec	Model 1	1.53 (0.85–2.76)	0.16
		Model 2	2.18 (0.96–4.98)	0.06
		Model 3	14.9 (2.45–91.0)	0.003
PI at 60 min^†^	Dec	Model 1	1.90 (1.02–3.56)	0.04
		Model 2	3.03 (1.27–7.22)	0.01
		Model 3	11.6 (2.46–54.5)	0.002
PI at 120 min^†^	Dec	Model 1	0.83 (0.47–1.45)	0.51
		Model 2	0.79 (0.39–1.62)	0.52
		Model 3	1.31 (0.56–3.08)	0.53
HOMA-R	Dec	Model 1	1.45 (0.81–2.58)	0.21
		Model 2	2.03 (0.91–4.53)	0.08
		Model 3	11.9 (2.13–66.2)	0.005
QUICKI	Inc	Model 1	1.73 (0.91–3.29)	0.10
		Model 2	2.67 (1.01–7.07)	0.048
		Model 3	17.3 (2.45–121)	0.004
HOMA-β	Dec	Model 1	1.79 (0.96–3.34)	0.07
		Model 2	2.62 (1.08–6.33)	0.03
		Model 3	22.7 (2.87–179)	0.003
Leptin	Dec	Model 1	0.76 (0.38–1.52)	0.43
		Model 2	0.88 (0.38–2.03)	0.77
		Model 3	2.45 (0.95–6.35)	0.06
TNFα	Inc	Model 1	1.54 (0.86–2.76)	0.15
		Model 2	1.76 (0.86–3.61)	0.12
		Model 3	1.57 (0.70–3.54)	0.27

Odds ratios for low serum amylase (< 60.0 U/ml) relative to normal-to-high serum amylase (≥ 60 Uml) are shown for each 1-SD increase/decrease in the clinical variables. All variables were log-transformed before analysis, except for QUICKI, FPG, and HbA1c.

Model 1: unadjusted.

Model 2: adjusted for age, sex, smoking, daily alcohol consumption, regular exercise, eGFR, systolic blood pressure, and high-density lipoprotein cholesterol.

Model 3: Model 2 plus adjustment for BMI.

*Per 1-SD increase (Inc) or 1-SD decrease (Dec).

†Measured during a 75-g OGTT.

*BMI*: body mass index; *FPG*: fasting plasma glucose; *FPI*: fasting plasma insulin; *HOMA*: homeostasis model assessment; *OGTT*: oral glucose tolerance test; *PG*: plasma glucose; *PI*: plasma insulin; *QUICKI*: quantitative insulin sensitivity check index; regular exercise: exercise over 30 minutes at least twice per week; *TNF*: tumor necrosis factor.

**Figure 1:**
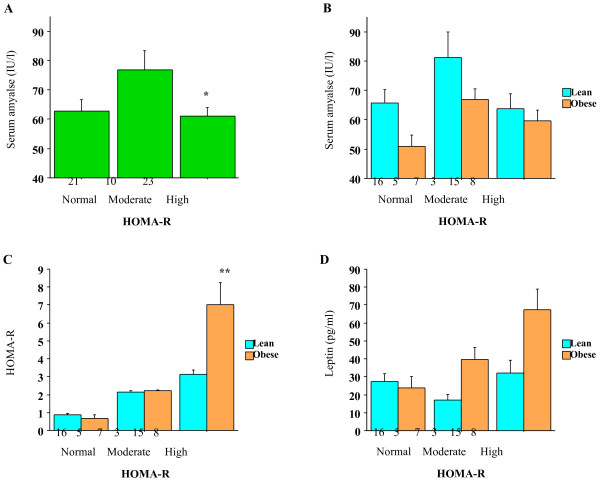
**A *.* Serum amylase levels according to HOMA-R.** HOMA-R was classified as normal (< 1.6), moderate (1.6–2.5), or high (> 2.5). The number of subjects in each group is shown under the bar. *Statistically significant difference between moderate and high HOMA-R (Bonferroni test, *P* = 0.014). Bars represent standard errors. B Serum amylase levels according to HOMA-R and obesity. Subjects were divided into three groups as in Figure 1A and then into lean (BMI<25 kg/m^2^) and obese (BMI ≥ 25 kg/m^2^) groups. The number of subjects in each group is shown under the bar. The difference between the lean and obese groups was of borderline significance (two-way ANOVA, *P* = 0.05). Bars represent standard errors. C HOMA-R according to HOMA-R and obesity. Subjects were divided as in Figure 1A and B*.* The number of subjects in each group is shown under the bar. HOMA-R was significantly different between lean and obese subjects in the high HOMA-R group (Mann–Whitney test, *P* = 0.001). Bars represent standard errors. D Leptin levels according to HOMA-R and obesity. Subjects were divided as in Figure 1A and B. The number of subjects in each group is shown under the bar. Bars represent standard errors.

When subjects were divided into three groups according to HOMA-R, the serum amylase levels in subjects with high HOMA-R were significantly lower than those in subjects with moderate HOMA-R (Bonferroni test, *P* = 0.014) (Figure 1A). When subjects were further divided into obese (BMI ≥ 25.0 kg/m^2^, n = 22) and lean (BMI<25.0 kg/m^2^, n = 32) groups, similar nonlinear trends were observed in both groups, which was of borderline significance in the obese group (two-way ANOVA, *P* = 0.05, Figure 1B). As shown in Figure 1C and D, no significant difference was observed in HOMA-R and leptin between the lean and obese groups, except for HOMA-R in the high HOMA-R group.

**Figure 2:**
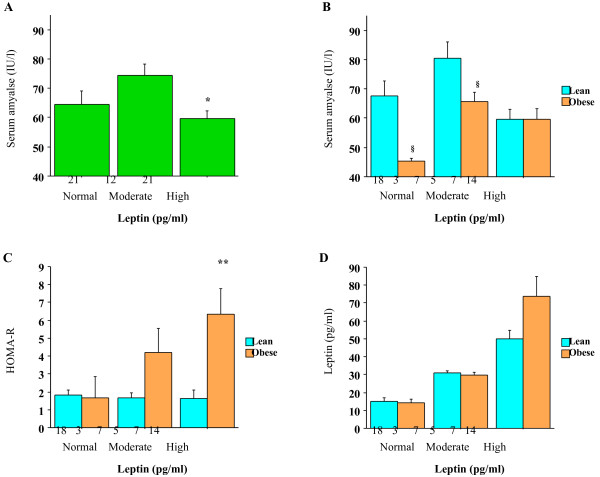
**A Serum amylase levels according to plasma leptin levels.** Subjects were divided into three groups based on leptin levels: normal (< 25 pg/ml), moderate (25–35 pg/ml), and high (> 35 pg/ml). The number of subjects in each group is shown under the bar. *Statistically significant difference between moderate and high leptin (Bonferroni’s test, *P* = 0.013). Bars represent standard errors. B Serum amylase levels according to leptin and obesity. Subjects were divided into three groups as in Figure 2A and then into lean (BMI<25 kg/m^2^) and obese (BMI ≥ 25 kg/m^2^) groups. The number of subjects in each group is shown under the bar. Leptin levels were significantly different between the lean and obese groups (two-way ANOVA, *P* = 0.02). ^§^Statistically significant difference between lean and obese subjects in the normal and moderate leptin groups (Mann–Whitney test, both *P*<0.05). Bars represent standard errors. C HOMA-R according to leptin and obesity. Subjects were divided as in Figure 2A and B. The number of subjects in each group is shown under the bar. HOMA-R was significantly different between lean and obese subjects in the high leptin group (Mann–Whitney test, *P* = 0.004). Bars represent standard errors. D Leptin according to the leptin and obesity. Subjects were divided as in Figure 2A and B. The number of subjects in each group is shown under the bar. Bars represent standard errors.

Similarly, as shown in Figure 2A, serum amylase levels in subjects with high leptin (> 35 pg/ml, n = 21) were significantly lower than those in subjects with moderate leptin (25–35 pg/ml, n = 12; Bonferroni test, *P* = 0.013). When subjects were divided into obese and lean groups, as above, similar trends were observed in both groups, and was significant in the obese group (two-way ANOVA, *P* = 0.02, Figure 2B). Additionally, serum amylase levels were significantly lower in obese subjects than in lean subjects among those with normal or moderate leptin levels, respectively (Mann–Whitney test, both *P*<0.05). As shown in Figure 2C and D, there were no significant differences in HOMA-R and leptin between the lean and obese subjects, except for HOMA-R in the high leptin group.

**Figure 3:**
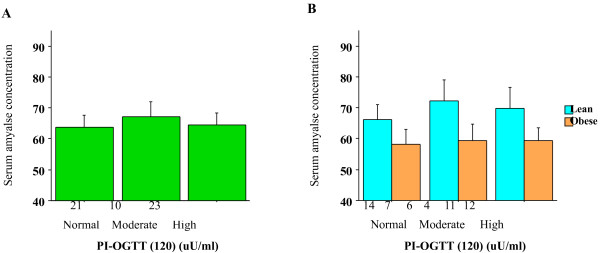
**A Serum amylase levels according to PI at 120 min during the OGTT.** Subjects were divided into three groups based on PI at 120 as normal (≤ 40 mg/dl), moderate (40–50.5 mg/dl), and high (> 50.5 mg/dl). The number of subjects in each group is shown under the bar. There were no significant differences among the three groups. Bars represent standard errors. B Serum amylase levels according to PI at 120 min during the OGTT and obesity. Subjects were divided as in Figure 3A and then into lean (BMI<25 kg/m^2^) and obese (BMI ≥ 25 kg/m^2^) groups. The number of subjects in each group is shown under the bar. The difference in PI at 120 min between the lean and obese groups was of borderline significance (two-way ANOVA, *P* = 0.06). Bars represent standard errors.

Similar trends were also observed when subjects were divided according to PI at 0 min (data not shown because of the robust similarities to Figure 1A and B). However, the trends in the nonlinear relationship were attenuated when subjects were divided according to PI at 120 min (Figure 3A), although borderline significant was observed between lean subjects and obese subjects (two-way ANOVA, *P* = 0.06, Figure 3B).

## Discussion

The present study showed a robust association between low serum amylase and BMI, as well as latent associations with decreased PI at 0 and 60 min, HOMA-R, and HOMA-β, and increased QUICKI in asymptomatic subjects without advanced diabetes. Notably, the later associations were not detected in earlier larger studies [3,5]. However, as in the previous studies, BMI was the most predominant factor explaining the serum amylase levels in linear and nonlinear manners. It is particularly interesting that adjustment for BMI and clinical confounders was necessary to unveil these latent associations in the multivariate logistic analysis. Indeed, this study revealed that, after adjusting for BMI, serum amylase was positively correlated with HOMA-R and FPI, which is attributable to the strong correlation between HOMA-R and FPI detected in this study. Since such high correlations are commonly observed in non-diabetic individuals [16,17], this finding is not contradictory.

### Nonlinear associations between low serum amylase and insulin resistance

The current results also showed complicated nonlinear associations between low serum amylase and high HOMA-R and high leptin (Figures 1 and 2), as low serum amylase was associated with severe insulin resistance but not moderate insulin resistance. A plausible explanation is that high insulin secretion and/or severe insulin resistance may cause insulin inactivity by downregulating insulin receptor expression or inhibiting insulin signaling in certain cells, including pancreatic acinar cells [18], eventually resulting in reduced insulinotropic action on the acinar cells [19]. By contrast, light to moderate insulin resistance is compensated for by increased insulin secretion, or moderately increased insulin secretion does not accompany insulin resistance, both of which lead to increased insulinotropic action and possibly increased serum amylase. Consistently, a similar nonlinear relationship was observed between serum amylase and circulating leptin levels. Increased circulating leptin, a marker for leptin resistance, is thought to be associated with insulin resistance [6,7] and metabolic syndrome [20].

It is noteworthy that similar nonlinear associations between serum amylase and HOMA-R and leptin were observed in obese individuals (Figures 1B and 2B), Furthermore, the significant difference in serum amylase between lean and obese subjects was particularly evident in the normal leptin group (Figure 2B). Similar trends were also observed in the normal HOMA-R group, albeit these were not significant (Figure 1B). Additionally, HOMA-R and leptin were broadly similar between lean and obese subjects in the normal leptin group and the normal HOMA-R group (Figures 1C and D, 2C and D). Then, although these trends should be confirmed in large studies because of small sample sizes, these findings suggest that other factors associated with high BMI, but not insulin resistance, may also contribute to the pathophysiology of low serum amylase in non-insulin-resistant individuals. As we proposed in a recent article [5], low serum amylase might reflect a physiological response to over-nutrition with reduced food absorption to regulate energy balance. Consistently, [Kondo et al. 21] showed that serum amylase was significantly lower in obese subjects than in lean subjects and that low serum amylase readily increased with diet therapy and weight loss, which commonly accompanies the improvement of insulin resistance, though. By contrast, the lower serum amylase levels in lean and obese subjects in the high leptin and high HOMA-R groups suggests that severe insulin resistance exceeding a threshold level may predict low serum amylase, independently of obesity. This might be related with a previous finding that insulin resistance can be associated with an increased risk of cardiovascular events independently of metabolic syndrome [22].

### Associations between low serum amylase and parameters in the fasting state

The observed associations of low serum amylase with HOMA-R, HOMA-β, PI at 0 and 60 min, but not with PI at 120 min, suggest that low serum amylase may be associated with low insulin action in the fasting state and for up to 1 h after feeding, rather than the later postprandial state. This finding may help to explain the significant association between serum amylase levels and FPG, but not with HbA1c, that was observed in previous large studies [3,5]. HbA1c is an important marker for overall circulating glucose levels, including the postprandial state. In this study, serum amylase was not associated with HbA1c, consistent with the earlier studies [3,5]. The lack of association between serum amylase and FPG in this study may be attributable to the small sample size.

Although TNFα was proposed as a candidate factor explaining the low serum amylase levels, unexpectedly, we found no significant association between low serum amylase and high TNFα in this study. This might be attributable to the small sample size and the specific characteristics of our study population, including the small proportions of subjects with overt diabetes, hypertension, or dyslipidemia. Alternatively, central insulin or leptin might be more effective on the serum levels of proinflammatory cytokines than systemic insulin or leptin, because a recent study by [Burgos-Ramos et al. 23] showed in animal study that intracerebroventricular administration of insulin and leptin remarkably reduced the serum levels of TNFα.

### Clinical relevance of low serum amylase

The clinical relevance of low serum amylase is still poorly understood. In clinical practice, a deficiency of pancreatic enzymes is known as pancreatic exocrine insufficiency (PEI), which is characterized by steatorrhea, malabsorption, and malnutrition [24,25]. PEI is commonly observed in individuals with pancreatic diseases, such as chronic pancreatitis and cystic fibrosis. However, PEI defined as low levels of fecal elastase-1 was found in approximately 50% of patients with type 1 diabetes and 20% of patients with type 2 diabetes [26,27]. These, and other results, indicate that low serum amylase may represent a close link between pancreatic endocrine dysfunction and exocrine dysfunction. Although it is unknown whether low serum amylase fully reflects PEI, it is possible that low serum amylase may be partially related with pathogenesis of PEI considering that insulin action is impaired in both type of diabetes. To fully elucidate this issue, further studies focusing on endocrine dysfunction and exocrine dysfunction are needed.

### Limitations

Some limitations of this should be mentioned. First, the sample size was small and the statistical power may be insufficient to reliably assess associations, particularly when the subjects were divided into sub-groups. Indeed, the large odds ratios and wide 95% confidence intervals, especially after adjustment for BMI, may be attributed to the small sample size. Second, the associations between variables were assessed in a cross-sectional manner, preventing us from determining causality. Prospective longitudinal studies and clinical trials are needed to evaluate the cause–effect relationship. Third, insulin resistance was assessed by HOMA-R, which is calculated from FPI and FPG. Ideally, insulin resistance should be assessed using specific methods, such as the hyperinsulinemic–euglycemic clamp test [28] or the frequently sampled intravenous glucose tolerance test [29]. However, because of the complexity of these tests, as well as their time-consuming procedures and expense, they were not performed in this study. It is also important to acknowledge that HOMA-R and QUICKI reflect hepatic insulin resistance rather than peripheral insulin resistance [30]. Thus, the current results should be interpreted with some caution. Finally, low serum amylase was defined as serum amylase < 60 IU/l, based on the results of an earlier large epidemiological study [3]. Different thresholds for low serum amylase are likely to yield different outcomes.

In conclusion, our results suggest a robust association between low serum amylase and BMI, as well as latent associations with decreased basal insulin and basal insulin secretion, and with insulin resistance in a nonlinear manner. These findings need to be confirmed in future large studies.

## Abbreviations

BMI, Body mass index; TNF, Tumor necrosis factor; OGTT, Oral glucose tolerance test; PG, Plasma glucose; PI, Plasma insulin; FPG, Fasting plasma glucose; FPI, Fasting plasma insulin; NGSP, National Glycohemoglobin Standardization Program; eGFR, Estimated glomerular filtration rate; HOMA-R, Homeostasis model assessment of insulin resistance; QUICKI, Quantitative insulin sensitivity check index; PEI, Pancreatic exocrine insufficiency.

## Competing interests

The authors declare that they have no competing interests.

## Authors’ contributions

TM, SM and MK (Kakei) designed the study; KN, AA, MY, HF, HM, SI and HS collected and analyzed the data; TM, KN, and MK (Kawakami) conducted literature reviews; and KN wrote the first draft of the manuscript. All authors reviewed and edited the manuscript, and approved the final version of the manuscript for publication.
